# Back Muscle Mass as a Predictor of Postoperative Complications in Posterior Lumbar Interbody Fusion Surgery †

**DOI:** 10.3390/jcm12165332

**Published:** 2023-08-16

**Authors:** Seung-Wan Hong, Ka-Young Rhee, Tae-Hoon Kim, Seong-Hyop Kim

**Affiliations:** 1 Department of Anesthesiology and Pain Medicine, Konkuk University Medical Center, Konkuk University School of Medicine, Seoul 05030, Republic of Korea; 20150077@kuh.ac.kr (S.-W.H.); rheeky@kuh.ac.kr (K.-Y.R.); 2 Deparment of Orthopedic Surgery, Konkuk University Medical Center, Konkuk University School of Medicine, Seoul 05030, Republic of Korea; 20060253@kuh.ac.kr; 3 Department of Medicine, Institute of Biomedical Science and Technology, Konkuk University School of Medicine, Seoul 05030, Republic of Korea; 4 Department of Infection and Immunology, Konkuk University School of Medicine, Seoul 05030, Republic of Korea

**Keywords:** muscle mass, postoperative complication, elderly

## Abstract

Background: There is a lack of studies on utilising skeletal muscle mass via preoperative lumbar computed tomography or magnetic resonance imaging as a predictor of postoperative complications of posterior lumbar interbody fusion (PLIF) surgery in elderly patients. Methods: Patients aged >65 years who underwent PLIF were enrolled. The sum of the cross-sectional areas of the erector spinae muscles (CSA_Both_) was presented as the skeletal muscle mass. Postoperative complications were assessed using CSA_Both_, pulmonary function testing, and prognostic nutritional index (PNI). Results: Patients with postoperative complications showed significantly lower values of CSA_Both_ (median 2266.70 (2239.73–2875.10) mm^2^ vs. 3060.30 (2749.25–3473.30) mm^2^, *p* < 0.001), functional vital capacity, forced expiratory volume at 1 s, and PNI. However, multiple logistic regression analysis identified American Society of Anaesthesiologists Physical Status (ASA PS) I (odds ratio 0.307 (95% confidence interval 0.110–0.852), *p* = 0.023), ASA PS III (4.033 (1.586–10.254), *p* = 0.003), CSA_Both_ (0.999 (0.999–1.000), *p* < 0.001), and postoperative red blood cell (RBC) transfusion (1.603 (1.193–2.152), *p* = 0.002) as risk factors for postoperative complications after PLIF surgery. Conclusions: CSA_Both_, ASA PS III, and postoperative RBC transfusion might be used as predictors of postoperative complications after PLIF in patients aged >65 years.

## 1. Introduction

The need for surgeries on elderly patients has increased with the increase in life expectancy. Although the physical condition of elderly patients and geriatric medicine have improved over time, concerns regarding morbidity or mortality after surgeries for elderly patients are the most critical factors influencing the decision to operate. Recently, several predictive tools for postoperative complications, such as preoperative pulmonary function testing (PFT) and the preoperative prognostic nutritional index (PNI), have been developed and utilised [1,2,3].

Spine surgery is the most common surgery performed on elderly patients. Notably, preoperative evaluation using lumbar computed tomography (CT) or magnetic resonance imaging (MRI) is essential to confirm the anatomical and functional status of the spine and the operation plan. Although preoperative lumbar CT or MRI are essential, there is a lack of studies on utilising skeletal muscle mass via preoperative lumbar CT or MRI as a predictor of postoperative complications of posterior lumbar interbody fusion (PLIF) surgery in elderly patients.

We hypothesised that back muscle mass might be associated with postoperative complications of PLIF surgery in elderly patients. This study aimed to evaluate the measurement of back muscle mass as a predictive tool for postoperative complications of PLIF surgery in elderly patients. We also evaluated the preoperative PFT and PNI as predictive tools for postoperative complications of PLIF surgery in elderly patients.

## 2. Materials and Methods

### 2.1. Study Population

The study was approved by the Institutional Review Board of Konkuk University Medical Center (approval number: KUH 2022-04-038) and registered at cris.nih.go.kr accessed on 3 July 2023 (registration number: KCT0007475). The data of the patients were obtained only from chart review, so written informed consent from the patients was not obtained. All data extracted from the chart review were de-identified before the analysis. Patients aged >65 years who underwent PLIF under general anaesthesia were enrolled from January 2016 to December 2021. Patients were excluded if any of the following criteria were met: (1) surgery for trauma, (2) pathologic fracture due to cancer, (3) surgery including thoracic or cervical lesion, (4) underlying inflammatory disease, (5) underlying cancer, or (6) other concurrent surgery.

### 2.2. Preoperative Lumbar CT or MRI

Lumbar CT or MRI was performed within 1 week before surgery. The images were obtained with a 2.5 mm thickness according to the institutional protocol.

**Figure 1:**
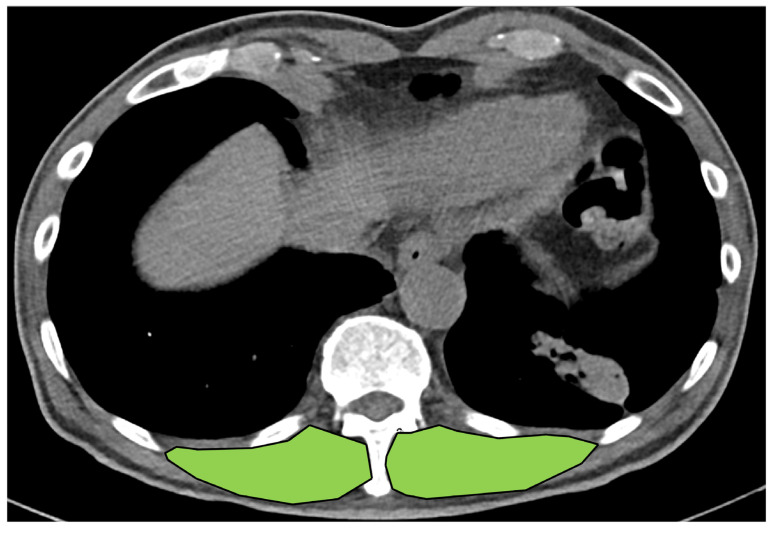
Measurement of total skeletal muscle mass in the erector spinae muscles at the level of the spinous process with the 12th thoracic vertebra. Erector spinae muscles are represented in green color.

To measure skeletal muscle mass, cross-sectional areas (CSAs) of the right and left erector spinae muscles (EMs) were measured. The contours of both EMs were identified at the level of the spinous process of the 12th thoracic vertebra. The CSAs of the right and left EMs were checked, and the sum (CSA_Both_) of the CSAs was presented as the skeletal muscle mass (Figure 1).

All procedures were performed by a radiologist who was blinded to the study, using the Centricity PACS Radiology RA1000 Workstation (GE Healthcare, Chicago, IL, USA).

### 2.3. Preoperative PFT

PFT was performed upon admission of the patient. Functional vital capacity (FVC), forced expiratory volume in the first second (FEV1), and the FEV1/FVC ratio were obtained.

### 2.4. Preoperative PNI

The PNI was calculated from the preoperative albumin and total lymphocyte counts in the blood. The formula of PNI was as follows: PNI = 10 × serum albumin (g/dL) + 0.005 × total lymphocyte count (/mm^3^) [4].

### 2.5. PLIF Surgery

All surgeries were performed by a single spine surgeon (THK) who had 10 years of experience. The patient was placed on the operating table in a prone position on a lumbar frame under general anaesthesia. A posterior midline incision was made in the skin and lumbosacral fascia. Posterior spinal structures, including the lamina and bilateral facet joints, were exposed. After inserting a pedicle screw instrument (Xia System; Stryker, Kalamazoo, MI, USA), total laminectomy and partial facetectomy were performed. After en bloc flavectomy, the thecal sac and transversing nerve roots were carefully removed to protect the nerve root retractor. The posterior annulus of the intervertebral disc was exposed and complete discectomy and endplate preparation were performed using a disc shaver, cage trial, and curved curette. After adequate decompression with the neural elements was performed, an autologous bone graft originating from the removed lamina or facet articular process was placed into the intervertebral space. Thereafter, two polyetheretherketone (PEEK) interbody cages (AVS UniLIF, Stryker, Kalamazoo, MI, USA) filled with autologous bone of appropriate size were bilaterally placed into the intervertebral space. Standard closure with the fascia and skin was performed.

### 2.6. Anaesthetic Technique and Intraoperative and Postoperative Management

Perioperative management was standardised, and the institutional protocol was followed. General anaesthesia was induced with propofol and remifentanil, and sevoflurane and remifentanil were used to maintain the anaesthesia. Anaesthetic depth, using state and response entropies between 40 and 50, was maintained under general anaesthesia. Rocuronium was administered for muscle relaxation during the monitoring of peripheral neuromuscular transmission. Haemodynamic stability within 20% of anaesthesia induction was maintained during anaesthesia. This neuromuscular blockade was reversed by sugammadex administration. Postoperative pain was controlled using an intravenous patient-controlled analgesia pump. Following the institutional protocol, the attending anaesthesiologist and the orthopaedic surgeon made the decision for postoperative admission to the post-anaesthetic care unit (PACU) or intensive care unit (ICU). Postoperative care in the general ward or ICU, except in the PACU for the attending anaesthesiologist, was provided by the orthopaedic surgeon, following the institutional protocol. Anaesthesia and operation time were assessed. Perioperative red blood cell (RBC) transfusions were reviewed.

### 2.7. Postoperative Complications after Spine Surgery during Hospitalisation

Postoperative complications, including neurologic, respiratory, cardiac, gastrointestinal (GI), hepatic, renal, and urinary tract infections, and wound complications during the hospital stay were evaluated. Deep vein thrombosis (DVT) was also evaluated. Postoperative complications were defined as grade III in the Clavien–Dindo classification [5].

Moreover, neurological complications such as delirium and cerebral infarction were also assessed. When any neurological symptoms or signs were not observed before anaesthesia, the orthopaedic surgeon checked them and consulted a neurologist or psychiatrist to assess, diagnose, and manage them. Delirium was diagnosed using the Confusion Assessment Method (CAM) [6], and if needed, a CT or MRI was performed.

Pulmonary complications such as atelectasis, pneumonia, pleural effusion, and pulmonary thromboembolism were evaluated. Postoperative chest radiography was performed in all patients. When new-onset abnormal findings on postoperative chest radiography or pulmonary symptoms or signs not observed before anaesthesia were observed, the orthopaedic surgeon checked them, performed chest CT, and consulted a pulmonologist to assess, diagnose, and manage them.

Cardiac complications such as haemodynamic instability and arrhythmia were assessed. Postoperative electrocardiography was performed in all patients. When cardiac symptoms or signs not observed before anaesthesia were observed, the orthopaedic surgeon checked them and consulted the cardiologist for assessment, diagnosis, and management.

GI and hepatic complications were evaluated. When GI or hepatic symptoms or signs not observed before anaesthesia were observed, the orthopaedic surgeon checked them and consulted with a gastroenterologist or hepatologist to assess, diagnose, and manage them.

Renal complications, including urinary tract infections, were evaluated using the Acute Kidney Injury Network criteria [7]. When renal symptoms or signs including urinary tract infection not observed before anaesthesia were observed, the orthopaedic surgeon checked them and consulted with a nephrologist to assess, diagnose, and manage them.

The presence of DVT was checked using routine CT evaluation 5 days after spine surgery.

Wound-related complications were also checked daily by the orthopaedic surgeon.

### 2.8. Statistical Analyses

Statistical analyses were performed using SPSS for Windows (version 27.0; IBM, Armonk, NY, USA). First, the demographic data were analysed according to the presence of postoperative complications. Categorical variables were analysed using the chi-squared or Fisher’s exact test, and continuous variables were analysed using an independent t-test. Second, univariate analysis for each potential risk factor for postoperative complications was performed using logistic regression. Factors with *p* < 0.05 in the univariate analysis were subjected to a multivariate conditional logistic regression model using the backward stepwise regression procedure. Odds ratios (ORs) with 95% confidence intervals (CIs) were calculated based on the final model. Third, the area under the curve (AUC) was calculated using receiver operating characteristic (ROC) curve analysis with Youden’s index to obtain cut-off values for statistically meaningful factors in predicting postoperative complications.

Data are expressed as the number of patients, mean ± standard deviation, or median (first-third quartile). Statistical significance was set at *p* < 0.05.

## 3. Results

**Figure 2:**
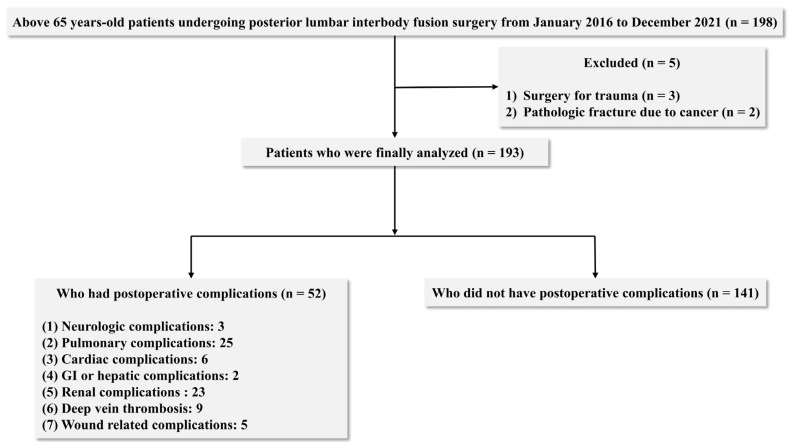
Flow chart for selection and classification of the patients.

During the study period, 198 patients aged >65 years underwent PLIF. Three patients who underwent surgery for trauma and two with pathologic fractures due to cancer were excluded. Finally, 193 patients met the inclusion criteria and were analysed (Figure 2).

Fifty-two patients developed postoperative complications. The most common postoperative complications were pulmonary and renal (Table 1).

Patients with postoperative complications had a significantly higher incidence of diabetes mellitus (DM) and pulmonary disease as underlying diseases. They also had a significantly lower incidence of ASA PS I and a significantly higher incidence of ASA PS III. Further, they had a significantly lower incidence of one-level PLIF surgery and required significantly more postoperative RBC transfusions (Table 2).

The hospitalisation duration of patients with postoperative complications was longer than that of patients without postoperative complications (25 ± 13 days vs. 16 ± 6 days, *p* < 0.001).

Patients with postoperative complications showed significantly lower values of CSA_Both_ in both genders (2266.70 (2239.73–2875.10) mm^2^ vs. 3060.30 (2749.25–3473.30) mm^2^, *p* < 0.001) (Table 3).

Univariate analysis identified DM, pulmonary disease, ASA PS I and III, CSA_Both_, FVC, FEV1, PNI, one-level PLIF surgery, and postoperative RBC transfusion as risk factors for postoperative complications after PLIF surgery. However, multiple logistic regression analysis identified ASA PS I [OR 0.307 (95% CI, 0.110–0.852), *p* = 0.023], ASA PS III [OR 4.033 (95% CI, 1.586–10.254), *p* = 0.003], CSA_Both_ [OR 0.999 (95% CI, 0.999–1.000), *p* < 0.001], and postoperative RBC transfusion [OR 1.603 (95% CI, 1.193–2.152), *p* = 0.002] as risk factors for postoperative complications after PLIF surgery (Table 4).

**Figure 3:**
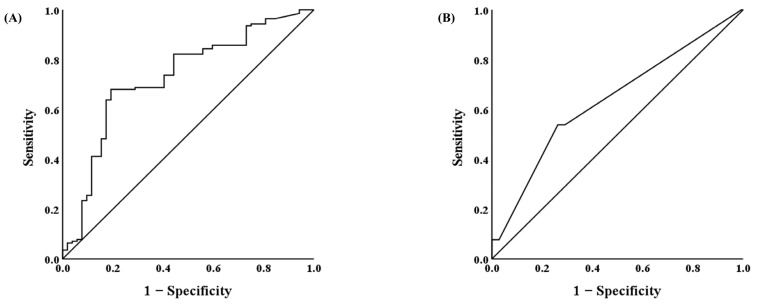
Receiver operating characteristic curve for the sum of cross-sectional area of the right and the left erector spinae muscles (CSA_Both_) (**A**) and postoperative red blood cell transfusion (**B**).

The AUCs for CSA_Both_ and postoperative RBC transfusion were 0.729 [95% CI, 0.646–0.811; *p* < 0.001] and 0.637 [95% CI, 0.545–0.728; *p* = 0.004], respectively (Figure 3).

The optimal threshold values for CSA_Both_ and postoperative RBC transfusion to predict postoperative complications based on ROC curve analysis were 2893.20 mm^2^ (sensitivity 68.1% and specificity 80.8%) for CSA_Both_ and 1.5 packs (sensitivity 53.8% and specificity 73.8%) for postoperative RBC transfusion.

## 4. Discussion

In the present study, pulmonary complications were the most common postoperative complications after PLIF surgery in patients aged >65 years. CSA_Both_, ASA PS III, and postoperative RBC transfusion were reliable predictors for postoperative complications.

Postoperative pulmonary complications have been reported in 6–80% of non-cardiothoracic surgeries. However, it depends on the definition, risk factors, severity of atelectasis, acute respiratory distress syndrome, and other factors [8]. The finding that the most common complications were pulmonary complications was anticipated because the patients enrolled in the present study were >65 years of age. Therefore, preoperative PFT was expected to be a reliable predictor of postoperative complications after PLIF surgery in patients, but it was insignificant in the present study. Although previous studies have demonstrated that PFT is a reliable predictor of postoperative complications in other surgeries [1,9,10,11,12], it has an innate limitation. For accurate evaluation of PFT, three factors should be considered: accurate instrumentation, a patient capable of performing tests, and skilled technologists [13], implying that patients’ understanding of PFT and effort during evaluation are essential for the exact evaluation of PFT. Particularly for elderly patients, performing PFT is difficult. Therefore, the PFT in the present study might have limited value.

The measurement of skeletal muscle mass has been demonstrated to be a reliable predictor of morbidity and mortality in patients undergoing spine surgery. Flexman et al. reported that sarcopenia is an important predictor of adverse outcomes after complex spinal surgery [14]. Hirase et al. also reported that sarcopenia predicts adverse events after revision surgery of the thoracolumbar spine [15]. However, the definition of sarcopenia, considering age, height, weight, etc., has not been established yet. Moreover, the heights and weights of the enrolled patients in the present study were not skewed. Therefore, CSA in the present study was used as an absolute value, not adjusted by age, height, weight, or other variables.

In the present study, CSA_Both_ showed higher sensitivity and specificity values of 68.1% and 80.8%, respectively, compared with postoperative RBC transfusion (sensitivity of 53.8% and specificity of 73.8%). This implies that CSA_Both_ was more effective in predicting postoperative complications after PLIF in patients >65 years of age compared to postoperative RBC transfusion.

We chose the CSA at 12th thoracic vertebra (T12) to measure back muscle mass for two reasons. First, skeletal muscle mass is independently associated with a decline in lung function [16]. EMs, a major anti-gravity muscle group, are known to reflect better physical activity than others [17]. Moreover, Asakura et al. revealed that CSA at T12 is significantly associated with pulmonary function and health-related quality of life [18]. Second, Fortin et al. demonstrated that EMs on the side of lumbar disc herniation are significantly smaller, although the degree of muscle asymmetry is not associated with symptom duration [19]. This implies that lesions on the spine might influence the muscle mass around the spine. The impact of muscle mass in elderly patients may be more important and may affect clinical outcomes. Therefore, CSA_Both_ may be associated with postoperative complications in patients aged >65 years undergoing PLIF.

Although the PNI has shown good predictability for adverse events after various surgeries [2,20], it did not significantly affect postoperative complications after PLIF surgeries in patients aged >65 years in the present study. The PNI has been developed for patients with cancer. Albumin level and total lymphocyte count were the dependent variables for PNI. By definition, the PNI is focused on nutrition. Although patients with cancer are likely to have low albumin and fewer lymphocytes, elderly patients undergoing PLIF surgeries in the present study were less likely to have these conditions. Considering that the chief complaints of elderly patients undergoing PLIF surgeries are back or leg pain and claudication [21], the patients had a low probability of malnutrition. Moreover, patients with trauma and a probability of malnutrition were not included in the present study.

The present study showed that ASA PS was a reliable predictor of postoperative complications after PLIF in patients aged >65 years. Recently, ASA PS has been reported to be a limitation in predicting postoperative complications in various studies [22,23]. The determination of ASA PS depends on the examiner’s subjective judgement [24]. Cassai et al. reported that ASA PS had a low to moderate inter-rater reliability in clinical practice, with a kappa value of approximately 0.38 [25]. However, the ASA PS is a simple metric that effectively stratifies risk evaluation for patients with different characteristics. Therefore, further evaluations should be conducted to determine the ASA PS value as a reliable predictor.

PLIF is an orthopaedic surgery that requires an intraoperative or postoperative RBC transfusion [26]. Notably, RBC transfusion is required for tissue damage. However, tissue damage caused by the transfusion itself leads to systemic inflammation. Ahmed et al. reported that patients with transfusion had higher rates of myocardial infarction, venous thromboembolism, wound infection, and mortality after cervical spine fusion than patients without transfusion [27]. Moreover, geriatric hip fracture surgery and postoperative transfusion have already demonstrated increased adverse outcomes, including postoperative pulmonary and cardiac complications, poor physical functioning recovery, longer hospital stays, and mortality in geriatric patients undergoing orthopaedic surgeries [28,29,30,31]. Therefore, postoperative transfusion might be a reliable predictor of postoperative complications after PLIF in patients aged >65 years.

The present study had a few limitations. First, the study was conducted retrospectively at a single centre. Therefore, the innate limitations of retrospective studies are inevitable. Second, the PLIF surgeries were performed by a single surgeon. Therefore, the results might have been biased. However, this might be a strength because selection bias was ruled out. The PLIF surgeries in the present study were performed under general anaesthesia. Notably, the results after surgery may vary according to anaesthesia type [32,33]. The results of the present study were limited to PLIF under general anaesthesia. Third, a radiologist who was blinded to the study performed the CSA measurement. Although previous studies have shown the predictive value of CSA from specific muscle mass measurement using CT images for postoperative complications without interobserver agreement [34], the interobserver agreement from two more radiologists in the present study might provide concrete results. Fourth, the gender effect might influence CSA_Both_. However, we did not consider the gender effect, because demographic data and univariate analysis did not differ according to gender. Moreover, the age of the enrolled patients was >65 years, with a limited influence of sex hormones.

In conclusion, CSA_Both_, ASA PS III, and postoperative RBC transfusion might be used to predict postoperative complications after PLIF in patients aged >65 years.

## Figures and Tables

**Table 1:** Postoperative complications after posterior lumbar interbody fusion (PLIF) surgery during hospitalisation.

Complications		Number of Patients
Neurologic complications		
	Delirium	3
	Cerebral infarction	0
Pulmonary complications		
	Atelectasis	10
	Pneumonia	12
	Pleural effusion	2
	Pulmonary thromboembolism	1
Cardiac complications		
	Hemodynamic instability	4
	Arrythmia	2
GI or hepatic complications		
	Hepatic enzyme elevation	2
Renal complications		
	Acute kidney injury	8
	Urinary tract infection	15
Deep vein thrombosis		9
Wound-related complications		5

Data are expressed as number of patients. Sixteen patients had two types of complications; five patients had three types of complications. Abbreviations: GI, gastrointestinal.

**Table 2:** Demographic data.

		With Cxs (*n* = 52)	Without Cxs (*n* = 141)	*p* Value
Gender (M/F)		22/30	48/93	0.289
Age (years)		74 ± 8	72 ± 7	0.090
Height (cm)		157 ± 7.0	158 ± 7.4	0.870
Weight (kg)		58 ± 11	59 ± 11	0.439
Underlying dz				
	HTN	29	60	0.102
	DM	27	46	0.014
	CVA history	3	7	0.492
	Pulmonary dz	11	10	0.005
	Cardiovascular dz	22	43	0.167
	Renal dz	7	13	0.113
Medication				
	ACEi or ARB	25	57	0.213
	β-blocker	10	15	0.138
	CCB	2	13	0.101
	Diuretics	11	22	0.363
	Hypoglycaemic	24	41	0.026
	Antiplatelet	17	45	0.221
ASA PS				
	I	6	47	0.006
	II	29	79	0.974
	III	17	15	<0.001
PLIF surgery level				
	1 level	8	45	0.022
	2 levels	30	60	0.061
	3 levels	11	32	0.819
	4 levels	2	3	0.505
	5 levels	1	1	0.460
Anaesthesia time (min)		304 (259–340)	284 (247–352)	0.965
Operation time (min)		251 (215–290)	229 (195–305)	0.833
RBC transfusion (pack)				
	Preoperative	-	-	-
	Intraoperative	0.00 (0.00–2.00)	0.00 (0.00–1.00)	0.235
	Postoperative	2.00 (0.00–2.00)	0.00 (0.00–2.00)	0.001
Hospitalisation (days)		25 ± 13	16 ± 6	<0.001

Data are expressed as number of patients, mean ± standard deviation or median (first-third quartiles). Abbreviations: With Cxs, with postoperative complications after PLIF surgeries; Without Cxs; without postoperative complications after PLIF surgeries; M, male; F, female; dz, disease; HTN, hypertension; DM, diabetes mellitus; CVA, cerebrovascular accident; ACEi, angiotensin-converting enzyme inhibitor; ARB, angiotensin receptor blocker; CCB, calcium channel blocker; ASA PS, American Society of Anaesthesiologists physical status; PLIF, posterior lumbar interbody fusion; RBC, red blood cell.

**Table 3:** Preoperative pulmonary function test (PFT) and the sum of cross-sectional area of the right and the left erector spinae muscles (CSA_Both_).

		With Cxs	Without Cxs	*p* Value
CSA_Both_ (mm^2^)		2666.70 (2239.73–2875.10)	3060.30 (2749.25–3473.70)	<0.001
Preoperative PFT				
	FVC (L)	2.81 (2.31–3.55)	3.23 (2.51–3.69)	0.033
	FEV1 (L)	2.13 (1.74–2.51)	2.43 (1.93–2.91)	0.002
	FEV1/FVC	76.00 (70.25–78.00)	76.00 (72.00–80.00)	0.295
PNI		47.18 ± 5.62	51.12 ± 7.37	0.001

Data are expressed as number of patients (%), mean ± standard deviation or median (first-third quartiles). Abbreviations: With Cxs, with postoperative complications after PLIF surgeries; Without Cxs; without postoperative complications after PLIF surgeries; CSA_Both_, sum of the cross-sectional areas of the right and left erector spinae muscles; PFT, pulmonary function test; FVC, functional vital capacity; FEV1, forced expiratory volume at first second; PNI, prognostic nutritional index.

**Table 4:** Predictors for postoperative complications after posterior lumbar interbody fusion (PLIF) surgery during hospitalisation.

Variables		Univariable		Multivariable	
		Hazard Ratio	*p* Value	Hazard Ratio	*p* Value
Gender		0.704 (0.367–1.350)	0.290		
Age		1.040 (0.994–1.088)	0.091		
Height		1.024 (0.884–1.334)	0.564		
Weight		1.113 (0.702–1.513)	0.448		
Underlying dz					
	HTN	1.565 (0.925–3.686)	0.480		
	DM	2.230 (1.167–4.264)	0.015		
	Cardiovascular disease	1.820 (0.856–3.465)	0.148		
	Pulmonary disease	3.515 (1.393–8.867)	0.008		
	CVA hx	1.665 (0.384–7.229)	0.496		
	Renal disease	2.667 (0.970–7.334)	0.150		
Current medications					
	ACEi or ARB	1.702 (0.897–3.232)	0.104		
	β-blocker	0.893 (0.543–1.160)	0.098		
	CCB	0.193 (0.025–1.514)	0.118		
	Diuretic	1.451 (0.648–3.250)	0.365		
	Hypoglycaemic agent	1.035 (0.477–2.249)	0.930		
	Antiplatelet agents	1.084 (0.786–2.044)	0.219		
ASA PS					
	I	0.261 (0.104–0.655)	0.004	0.307 (0.110–0.852)	0.023
	II	1.011 (0.533–1.917)	0.974		
	III	4.080 (1.854–8.980)	<0.001	4.033 (1.586–10.254)	0.003
CSA_Both_		0.999 (0.998–0.999)	<0.001	0.999 (0.999–1.000)	<0.001
Preoperative PFT					
	FVC (L)	0.635 (0.435–0.927)	0.019		
	FEV1 (L)	0.374 (0.204–0.686)	0.001		
	FEV1/FVC	0.995 (0.960–1.031)	0.785		
PNI		0.917 (0.871–0.965)	0.001		
PLIF surgery level					
	1 level	0.388 (0.169–0.892)	0.026		
	2 levels	1.841 (0.967–3.504)	0.063		
	3 levels	0.914 (0.422–1.981)	0.819		
	4 levels	1.840 (0.299–11.336)	0.511		
	5 levels	0.364 (0.022–5.933)	0.478		
Anaesthesia time		1.000 (0.995–1.005)	0.892		
Operation time		1.000 (0.995–1.005)	0.993		
RBC transfusion					
	Preoperative	-	-		
	Intraoperative	0.762 (0.539–1.078)	0.124		
	Postoperative	1.554 (1.210–1.996)	0.001	1.603 (1.193–2.152)	0.002

Abbreviations: dz, disease; HTN, hypertension; DM, diabetes mellitus; CVA, cerebrovascular accident; ACEi, angiotensin-converting enzyme inhibitor; ARB, angiotensin receptor blocker; CCB, calcium channel blocker; ASA PS, American Society of Anaesthesiologists physical status; CSA_Both_, sum of cross-sectional area of right and left erector spinae muscles; PFT, pulmonary function test; FVC, functional vital capacity; FEV1, forced expiratory volume at first second; PNI, prognostic nutritional index; PLIF, posterior lumbar interbody fusion; RBC, red blood cell.

## Data Availability

The datasets are available from the corresponding author on reasonable request.
